# A Novel Technique for Constructing Infectious Cloning of Type 3 Porcine Circovirus

**DOI:** 10.3389/fmicb.2020.01067

**Published:** 2020-06-09

**Authors:** Zaixue Jiang, Jiajun Wu, Mei Jiang, Yilun Xie, Wandi Bu, Canbin Liu, Guihong Zhang, Manlin Luo

**Affiliations:** Key Laboratory of Zoonosis Prevention and Control of Guangdong Province, College of Veterinary Medicine, South China Agricultural University, Guangzhou, China

**Keywords:** porcine circovirus type 3, sequence rearrangement, cyclized PCV3 DNA, PCV3 infectious cloning, cellular immunofluorescence

## Abstract

Porcine circovirus type 3 (PCV3), which currently lacks effective preventive measures, has caused tremendous economic losses to the pig husbandry. Obtaining the strain of PCV3 is the key to preparing related vaccines and developing corresponding antiviral drugs. In this study, according to the linear sequence of PCV3 DNA published on GenBank, the sequence was rearranged with SnapGene gene-editing software, and after rearrangement, the *Hin*dIII restriction endonuclease site was added to the end of the linear DNA, so that both ends have the same restriction endonuclease site. On this basis, the rearranged linear DNA is obtained by gene synthesis, PCR amplification, DNA purification, etc., and is digested and connected *in vitro* to obtain cyclized DNA. PCV3 infectious clones were obtained by transfecting 3D4/21 cell lines. The obtained PCV3 was identified by PCR, Western blotting, and indirect immunofluorescence tests. The results showed that this study successfully obtained the strain of PCV3 *in vitro*. To further evaluate the pathogenicity of the obtained PCV3 infectious clones, this study established an animal model of Kunming mice infected with PCV3. The results of RT-PCR, Western blotting and immunohistochemistry showed that PCV3 can infect myocardium and alveoli of Kunming mice, but no PCV3 was detected in other tissues. The above studies indicate that PCV3 circular DNA can be used to construct PCV3 infectious clones. This research will provide a new method for the construction of circular DNA viruses and lay the foundation for the research and pathogenesis of PCV3 vaccine.

## Introduction

Porcine circovirus has long harmed the sound development of pig husbandry, and especially the PCV3 that has appeared in recent years has caused tremendous economic losses for pig husbandry (Li and Tian, 2017; Ouyang et al., 2019). Current research indicates that PCV is mainly divided into three genotypes (Ge et al., 2018). PCV1 is generally considered non-pathogenic (Tischer et al., 1986). PCV2 is widely prevalent worldwide, previous studies have shown that PCV2 is the main pathogenesis of post-weaning multisystemic wasting syndrome (PMWS) and swine dermatitis and nephrotic syndrome (PDNS) (An et al., 2007; Welti et al., 2012). However, in recent years, some researchers have detected PCV3 from PDNS piglets (Palinski et al., 2017; Kedkovid et al., 2018). Studies have shown that PCV3 and PCV2 are mixed infections and have become popular in many countries. It has been reported that PCV3 may cause reproduction disorder in sows and PDNS in adult pigs (Palinski et al., 2017). Similar to PCV2, PCV3 is often mixed with PRRSV, PCV2 (Chen et al., 2019). However, unlike PCV2, there are currently commercial vaccines to prevent PCV2 infection (Park et al., 2017), and there is still a shortage of vaccines and related drugs to prevent PCV3.

Obtaining standard virus strains is the basis for the development of PCV3 vaccines and related biological products. However, no experimental report on the successful isolation of PCV3 has been reported. With the development of genetic engineering technology, a variety of virus strains have been constructed through reverse genetics (Yoo et al., 2004; Huang et al., 2011). With the continuous analysis of the genome structure and function of PCV2 and PCV3, researchers have obtained infectious clones by constructing eukaryotic expression vectors of PCV2 and PCV3 (Wang et al., 2015; Jiang H. et al., 2019). According to the genome structure of PCV, the porcine circovirus genome is a single-stranded negative-stranded DNA, the virions are only 14–17 nm, and there is no capsule on the surface of the virus capsid (Mankertz et al., 2004; Xiao et al., 2015). The size of the PCV1 and PCV2 genome research surface is 1767–1768 bp and contains 11 open reading frames (ORFs), of which ORF1 is a virus replication–related protein (Rep) and is a necessary element of virus replication (Cheung, 2012). ORF2 encodes the viral capsid protein (Cap) (Nawagitgul et al., 2000) and is commonly used in the study of subunit vaccines and diagnostic reagents (Li et al., 2017). Unlike the genomes of PCV1 and PCV2, the full length of the PCV3 genome is 2000 bp (Phan et al., 2016). Some researchers have predicted the PCV3 genomic DNA, and currently they have predicted a total of three ORFs (Palinski et al., 2017). One Rep protein composed of 297 amino acids was encoded by ORF1 (Ye et al., 2018). Besides, another Cap protein covering 214 amino acids by ORF2 was replicated in the opposite direction and a protein with an unknown function and containing 231 amino acids by ORF3 (Faccini et al., 2017). Whether PCV3 has other open reading frames and their functions still needs further study.

The above research is to construct infectious clones under the premise of grasping the genome structure of the virus. However, for some newly discovered circular DNA viruses, the genome structure and function are undefined yet, so it is hard to construct infectious clones with the application of the eukaryotic expression vector. This study intends to use the biological characteristics of PCV3 circular DNA to construct PCV3 infectious clones and construct a Kunming mouse infection model without the help of exogenous expression vectors. The results showed that the PCV3 strain can infect the myocardium and lung of mice. This study will provide a method for the construction of infectious clones of other circular DNA viruses and lay a foundation for the study of the pathogenic mechanism of PCV3.

## Materials and Methods

### Cells and Cultures

The 3D4/21 cell line (iCell Bioscience Inc., Shanghai, China) was cultivated in Dulbecco’s minimum essential medium (MEM, Gibco) supplemented by 10% fetal bovine serum at 37°C in a humidified 5% CO_2_ incubator. The cloning and construction of recombinant expression plasmids was carried out in *E. coli* strain DH5α cells (Takara Bio, Dalian, China). The prokaryotic expression vector pET-32a (+) and *E. coli* BL21 (DE3) cells were harvested from the stocks of our laboratory. The SP2/0 cells were also acquired from the stocks.

### Viral Gene and Primer Synthesis

**FIGURE 1:**
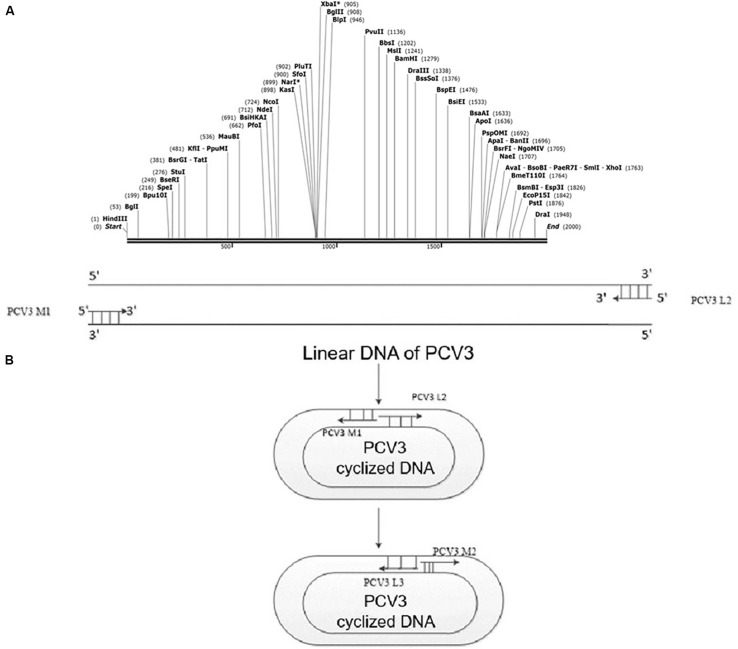
PCV3 viral gene rearrangement. **(A)** Rearranged linear gene sequence of PCV3 containing *Hin*dIII restriction sites. The *Hin*dIII restriction site acted as the reopening site, added to the end of the linear sequence after the opening process. **(B)** PCR detection after PCV3 cyclization. Specific primers, covering PCV3 M2 and PCV3 L3, were designed to verify whether the PCV3 gene sequence was cyclized. However, the PCV3 linear sequence could not be amplified by the mentioned primers.

The PCV3 gene sequence (GenBank accession number: MH107162.1) was taken to form a loop on Snap-Gene software, and the position of the unique restriction site was adopted to open the sequence, termed as the rearranged linear PCV3 gene sequence. Since the PCV3 is circular, the cyclization of the original linear DNA sequence was considered, the *Hin*dIII restriction site was recruited as the reopening site. When the opening process was completed, the *Hin*dIII restriction site was added to the end of the linear sequence. The newly rearranged sequence was synthesized by Sangon (Shanghai, China). Figure 1 illustrates the gene pattern of the novel PCV3 and presents the design pattern of the circular PCV3 amplification primer and detection primer. Primers listed in Table 1 were designed in accordance with the rearranged PCV3 gene sequence and PCV3 Cap gene sequence; the designed primers were synthesized by Sangon (Shanghai, China).

**TABLE 1:** Primers used for the construction and identification of the recombinant virus.

Primer Sequence
PCV3 M1: 5′-CCCAAGCTTGTGCGGATGCGGCTGCGCG-3′;
PCV3 L2: 5′- CCCAAGCTTCCCGCGTTTTCCCACAACC-3′;
PCV3 L3: 5′- GGTTGTGGGAAAACGCGGG-3′;
PCV3 M2: 5′-CGCGCAGCCGCATCCGCACAAGCTT-3′;
PCV3 Cap *Bam*HI: 5′-CGCGGATCCATGAGACACAGAGCTATATTCAGAA-3′;
PCV3 Cap *Hin*dIII: 5′-CCCAAGCTTTTAGAGAACGGACTTGTAACGAATC-3′

*The underlined sequences represent restriction enzyme sites introduced. PCV3 M1, PCV3 L2: PCV3 complete genome amplification primers; PCV3 L3 is a reverse complementary sequence of PCV3 L2; PCV3 M2 is the nucleotide sequence next to the primer binding site of PCV3 L2; PCV3 Cap BamHI, PCV3 Cap HindIII: The design of the primer is the reverse complement of the negative chain of PCV3 with the Cap gene sequence, and specific primers were then designed according to the reverse complementary DNA sequence.*

### Amplification and Cyclization of the PCV3 Genome

To amplify the newly synthesized rearranged PCV3 gene, PCV3 M1, PCV3 L2 primers, and KFX-101 high fidelity enzymes (TOYOBO, Japan) were adopted. The final volume of reaction fluid reached 100 μL. The PCR cycling conditions included: pre-denaturation at 94°C for 2 min, 30 cycles at 98°C for 10 s, 58°C for 30 s, and 68°C for 2 min, as well as the final extension for 10 min at 68°C. PCR products were preserved at 16°C for subsequent experiments.

The amplified PCR products of rearranged PCV3 gene were purified following the instructions of the DNA Purification Kit (Takara Bio, Dalian, China). Subsequently, the purified PCR product was digested by *Hin*dIII restriction endonuclease (NEB, Beijing, China) and then incubated in a water bath at 37°C for 5 h. Then, the digested products of *Hin*dIII restriction endonuclease were purified following the manufacturer’s instructions (Takara Bio, Dalian, China). Next, the purified DNA fragments were connected based on T4 DNA ligase instructions (NEB, Beijing, China) at 16°C for 12 h. Last, the cyclized PCV3 DNA was harvested.

### Transfection of Cyclized PCV3 DNA

The cyclized PCV3 DNA was transfected into 3D4/21 cells (60% confluency) in each well of the 6-well plates. Next, cells were transfected with of 10 μg cyclic PCV3 DNA with the use of Lipofectamine 3000 (Invitrogen, Carlsbad, CA, United States) following the manufacturers’ protocol. Afterward, the mixture was gently blended with a pipette and cultivated in a 5% carbon dioxide incubator at 37°C for 7–10 days.

### Detection of PCV3 Infectious Clone by PCR

Nucleic acid was extracted from cyclized PCV3 DNA transfected cells and normal cell supernatant in accordance with the instructions of the Takara virus DNA/RNA Extraction Kit (Takara Bio, Dalian, China) to perform PCR and RT-PCR. PCV3 L3, PCV3 M2 primers and Premix Taq^TM^ (Takara Bio, Dalian, China) were adopted for the common PCR detection, and PCV3 Cap *Bam*HI and PCV3 Cap *Hin*dIII primers and a PrimeScript^TM^ One Step RT-PCR Kit (Takara Bio, Dalian, China) were employed to perform the RT-PCR analyses.

The schemes of PCR cycling included: 94°C for 4 min, 30 cycles at 94°C for 30 s, 58°C for 30 s, and 72°C for 2 min, as well as the final extension for 10 min at 72°C. The RT-PCR procedure included: reverse transcription at 50°C for 30 min, pre-denaturation at 94°C for 4 min, 30 cycles at 94°C for 30 s, 58°C for 35 s and 72°C for 2 min, as well as the final extension at 72°C for 10 min.

### Preparation of Specific Monoclonal Antibody Against PCV3 Cap

To produce monoclonal antibodies (mAbs) against PCV3 Cap, recombinant PCV3 Cap protein was expressed with the prokaryotic expression system. In brief, PCV3 Cap gene was amplified and then cloned into pET-32a (+) vector; subsequently, it was expressed in *E. coli* and then purified with a His-tag protein purification kit (CWBio, Beijing, China). Likewise, PCV1 and PCV2 Cap protein were prepared and then purified.

Five 6-week-old female BALB/c mice underwent the subcutaneous injection of 50 μg recombinant PCV3 Cap and 50 μL Freund’s complete adjuvant in a final volume of 100 μL. Three booster immunizations, each of which had the equivalent dosage of antigen plus Freund’s incomplete adjuvant, were administered at the intervals of 2 weeks. Five days after the final booster injection, blood samples were collected via the tail vein. Subsequently, the mice were euthanized, and the spleen was obtained and then fused with SP2/0 myeloma cells to screen out the positive hybridoma cells as previously described (McNeilly et al., 2001). The mice were injected in their abdominal cavity with positive hybridoma cells secreting anti-PCV3 Cap antibodies. The ascites of the mice were collected after the occurrence of abdominal bulge; then, they were filtered and purified. The anti-PCV3 Cap mAb was obtained and named after the hybridoma cell line. PCV3 Cap, PCV2 Cap, PCV1 Cap, and His-tagged proteins acted as detection agents, and hybridoma cells were screened by indirect ELISA.

### Western Blot

The reactivity of mAbs to recombinant PCV3 Cap and rescued PCV3 was ascertained by a Western blotting assay. The virus and protein were fractionated on a 10% Sodium Dodecyl Sulfate-Polyacrylamide Gel Electrophoresis (SDS-PAGE) gel; next, they were transferred to PVDF membrane and then blocked with PBST buffer supplemented by 5% skim milk powder for 2 h. Subsequently, PCV3 Cap mAb (1/1000 dilution in PBST) was adopted as the primary antibody, and goat anti-mouse HRP-IgG (1/5000 dilution in 5% skim milk blocking solution) acted as the secondary antibody after rinsing five times with PBST. Lastly, the color development of PVDF membranes was achieved following the instructions of DAB color development kit (CWBio, Beijing, China).

### Indirect Immunofluorescence Assay

3D4/21 cells were cultivated in 6-well cell culture plates. When 70% of the cells were fused, 200 μL rescued PCV3 virus solution was added, and the cells without inoculation were set as the control. After 72 h cell culture, the supernatant was abandoned, and the cells were subsequently rinsed with PBS. In each well, 500 μL 4% paraformaldehyde (PFA) was added to fix cells at ambient temperature for 10 min. Then, 200 μL 0.1% Triton X-100 was applied for rinsing at ambient temperature for 10 min, and 500 μL 3% BSA solution was applied for blocking at ambient temperature for another 1 h. The ascites identified as positive by a Western blotting assay acted as the first antibody (1/200 dilution in PBS), and goat anti-mouse TRITC-IgG acted as the secondary antibody (1/1000 dilution in PBS) to incubate cells. Lastly, a DAPI staining kit (Beyotime, Shanghai, China) was employed to observe the cell morphology as irradiated by the red excitation light.

### Viral Titration for Serial Passages

Virus titers in cell cultures for each passage were measured by IFA, and TCID_50_/ml was recorded with Reed–Muench method, and then draw virus growth curve. The viral cultures were serially diluted 10-fold in DMEM medium supplemented by 5% FBS and antibiotics. The rescued PCV3 virus dilution was inoculated into 96-well plates covering 100 μL 3D4/21 cells in suspension. After the incubation for 2 h at 37°C, the liquids in 96-well plates were removed, and DMEM with 2% FBS was added to the wells. Subsequently, the plates were incubated for an additional 72 h at 37°C.

### Animal Experimental Design

Two experiments were designed to analyze the pathogenesis of PCV3 infection in mice. Ten 6-week-old KM mice were randomly split into two groups. The mice in the experimental group were intraperitoneally injected with the 0.5 mL rescued PCV3 virus, respectively. The mice in the control group were inoculated with the identical dose of PBS. Each group of mice was housed in an individual room while fed sterile food and water. Mice were subject to euthanasia 21 days after infection, and then tissue samples were dissected and collected. If the mice died in the experiment, they would be immediately dissected and their tissue samples would be collected.

### Clinical and Pathological Examination

The clinical symptoms of mice infected with virus were observed every morning and evening. After 21 days of the infection, the mice were dissected, and the pathological variations of various tissues and organs were recorded. The tissues of heart, liver, spleen, lung, kidney, brain, and lymph nodes were fixed with 4% formaldehyde solution. Paraffin sections were produced routinely, stained with HE, and subsequently observed under a microscope.

### Immunohistochemical Staining

To detect PCV3 viral antigen, the histopathological tissue samples were stained with IHC. The tissue samples were harvested from PCV3-inoculated mouse. First, the tissue samples were fixed with 4% formaldehyde to develop paraffin blocks. The slices were cut into 3–4 μm thick slices and then incubated overnight at 37°C. Subsequently, the slides were de-waxed and then blocked with 3% H_2_O_2_ for 10 min at ambient temperature. After rinsing with PBS five times, the slides were incubated with normal goat serum (1/20 dilution in PBS) at 37°C for 15 min and subsequently with PCV3 Cap mAb (1/100 dilution in PBS) at 4°C overnight. Afterward, the slides were rinsed with PBS and then cultivated with goat anti-mouse HRP-IgG (1/200 dilution in PBS) at 37°C for 1 h before being counterstained with hematoxylin for 10 s. Last, the slides were analyzed under a microscope.

### Statistical Analysis

All the experiments data are expressed as the mean ± Standard Deviation (SD). The statistical analysis was conducted by Student’s *t* test, and *p* value < 0.05 was considered as to be of statistical significance.

## Results

### Construction of PCV3 Infectious Clone

**FIGURE 2:**
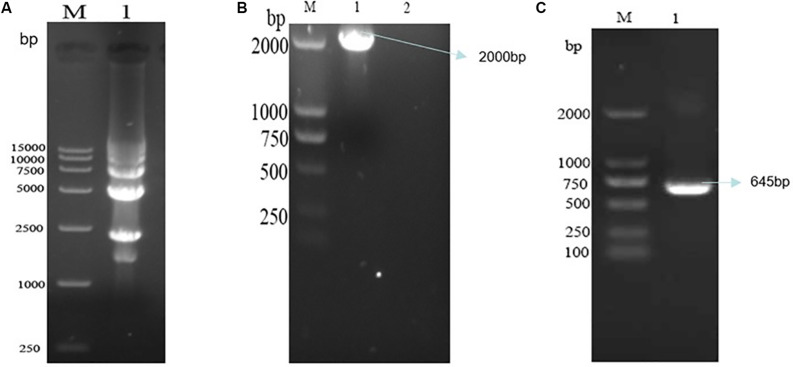
The detection of cyclized PCV3 DNA and the screening of recombinant plasmids. **(A)** Electrophoresis results of PCV3 DNA nucleic acid after cyclization. M, DL15000 DNA Marker; Lane 1, cyclized PCV3 DNA. **(B)** Nucleic acid electrophoresis results of cyclized PCV3 DNA common PCR products, and the size of the amplified PCR products were 2000 bp. M, DL2000 DNA Marker; Lane 1, PCR products of the supernatant of transfected cells; Lane 2, PCR products of the supernatant of normal cells. **(C)** Nucleic acid electrophoresis results of cyclized PCV3 DNA RT-PCR products, and the size of the amplified PCR products were 645 bp. M, DL2000 DNA Marker; Lane 1, RT-PCR products of the supernatant of transfected cells.

The PCV3 infectious clone was achieved by self-cyclization of the genomic DNA followed digestion of recombinant genomic DNA by *Hin*dIII, gel extraction and ligation at the created *Hin*dIII site by T4 DNA ligase (Figure 2A). The identified cyclized PCV3 DNA was transfected into the 3D4/21 cell line with Lipofectamine 3000 (Invitrogen) in accordance with the manufacturer’s protocol.

Nucleic acid was extracted from the rescued viruses analyzed by PCR and RT-PCR. The full-length (2000 bp) PCV3 PCR products were amplified from the DNA extracted from the rescued cells (Figure 2B). The PCV3 Cap gene length of the RT-PCR products amplified by primers PCV3 Cap *Bam*HI and PCV3 Cap *Hin*dIII reached 645 bp (Figure 2C). The identical bands of the expected sizes were achieved; it is therefore revealed that the cyclized PCV3 DNA was detected, and the PCV3 Cap gene was replicated and then transcribed.

### Identification and Potency Analysis of PCV3 Monoclonal Antibody

**FIGURE 3:**
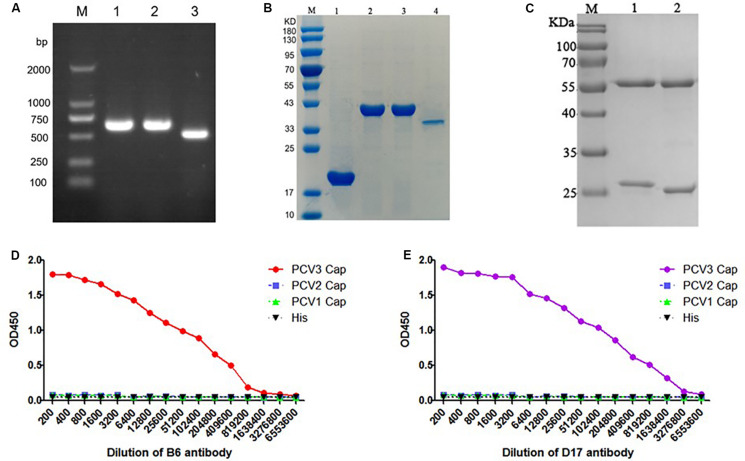
**(A)** PCR nucleic acid electrophoresis of *E. coli* BL21 (DE3) consisting of the pET-32a-PCV Cap plasmid, and the destination fragments were amplified by PCR. M, DL2000 DNA Marker; Lane 1, PCR nucleic acid electrophoresis of pET-32a-PCV1 Cap in *E. coli* BL21 (DE3); Lane 2, *E. coli* BL21/pET-32a-PCV2 Cap; Lane 3, *E. coli* BL21/pET-32a-PCV3 Cap. **(B)** Purification of the PCV3 Cap protein and SDS-PAGE electrophoretic results of purified PCV Cap protein and the size of the bands were 40, 40 and 35 kDa, respectively. M, Protein Marker; Lane 1, BL21 (DE3)/pET32a purified protein sample; Lane 2, BL21 (DE3)/pET32a-PCV1 Cap purified protein sample; Lane 3, BL21 (DE3)/pET32a-PCV2 Cap purified protein sample; Lane 4, BL21 (DE3)/pET32a-PCV3 Cap purified protein sample. **(C)** The SDS-PAGE results of purification monoclonal antibody. M, Protein Marker; Lane 1, B6 mAb; Lane 2, D17 mAb. **(D)** The potency analysis titer curve of purified B6 mAb. **(E)** The potency analysis titer curve of purified D17 mAb.

The figure reveals the successful building of recombinant plasmid pET32a-PCV1 Cap, pET32a-PCV2 Cap, pET32a-PCV3 Cap (Figure 3A). Meantime, recombinant PCV1, PCV2, and PCV3 Cap proteins exhibited successful expression using prokaryotic expression system; then, they were purified (Figure 3B).

The indirect ELISA method was employed to ascertain the titer of mouse serum and anti-PCV3 Cap protein mAb. As suggested from the results, the titer of mouse serum Number 1 was the optimal, reaching over 1:64,000. The spleen cells of that mice were fused. Seven days later, unfused cells showed the lysis and death, and grape clusters were formed in fused cells. Last, through reactions with PCV1, PCV2, and PCV3 Cap proteins, two specific PCV3 Cap hybridoma cell lines were achieved after triple cloning, purification and screening. The cell lines were nominated as B6 and D17. Table 2 lists the indirect ELISA results for the supernatants of hybridoma cells.

**TABLE 2:** Screening results of ELISA for positive hybridoma cells.

Number	PCV3 Cap	PCV2 Cap	PCV1 Cap	His
1	0.11	0.052	0.04	0.732
2	0.021	0.039	0.07	0.052
3	0.057	0.037	0.053	0.867
4	0.62	0.509	0.317	0.017
5	0.029	0.022	0.019	0.016
6	0.851	0.052	0.033	0.025
7	0.071	0.026	0.015	0.055
8	0.509	0.221	0.156	0.027
9	0.341	0.309	0.281	0.011
10	0.015	0.016	0.012	0.067
11	0.023	0.015	0.025	0.033
12	0.029	0.028	0.033	0.867
13	0.047	0.029	0.023	0.015
14	0.038	0.024	0.021	0.032
15	0.019	0.028	0.014	0.917
16	0.044	0.036	0.023	0.017
17	0.717	0.016	0.011	0.018
18	0.016	0.012	0.001	0.835
19	0.016	0.023	0.015	0.649
20	0.044	0.036	0.023	0.017

*The blue font is the result of an ELISA reaction with only the PCV3 Cap.*

As suggested from SDS-PAGE results of the two purified antibodies, the antibody was well purified, and the antibody had obvious light and heavy chains (Figure 3C). Meantime, the antibody titer results suggested that the B6 mAb titer was 2 × 10^5^ (Figure 3D), and the D17 mAb titer was 6 × 10^5^ (Figure 3E). According to the results of antibody subclass identification, the B6 mAb was IgG1, and the D17 mAb was IgG2b (Table 3).

**TABLE 3:** Analysis of monoclonal antibody subtypes.

Number	6	17
M	0.051	0.038
G1	0.155	0.001
G2a	0.018	0.027
G2b	0.009	0.230
G3	0.016	0.018
A	0.016	0.015
κ	0.045	0.031
λ	0.034	0.033

*Blue font is positive reaction results.*

### Western Blotting Assay

**FIGURE 4:**
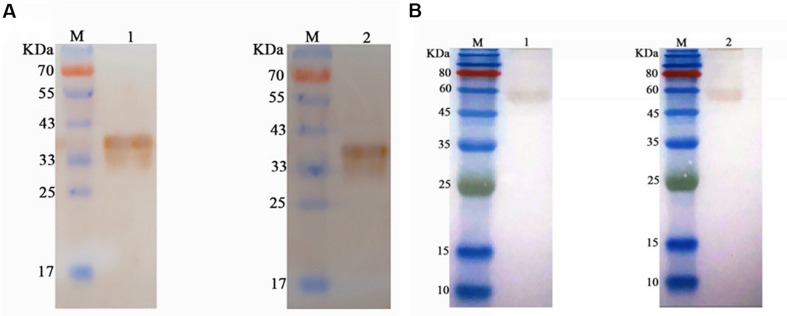
Western blotting assay of B6 mAb and D17 mAb. **(A)** Western blotting assay of the purified PCV3 Cap protein, suggesting the specific band at 35 kDa. M, Protein Marker; Lane 1, Western blotting assay of the purified PCV3 Cap samples using B6 mAb; Lane 2, Western blotting assay of the purified PCV3 Cap samples using D17 mAb **(B)** Western blotting assay of the rescued PCV3, and the size of the bands on the PVDF membrane were approximately 60 kDa. M, Protein Marker; Lane 1, Western blotting assay of the rescued PCV3 using B6 mAb; Lane 2, Western blotting assay of the rescued PCV3 using D17 mAb.

The results of the Western blotting assays of anti-PCV3 Cap protein mAb demonstrated that the purified recombinant PCV3 Cap protein displayed a specific band at 38 kDa. The anti-PCV3 Cap protein mAb could react with PCV3 Cap to reach the target band (Figure 4A), suggesting that the PCV3 Cap obtained here exhibited prominent immunogenicity. The rescued PCV3 virus was tested by Western blotting assays with D17 mAb. The size of bands on the PVDF membrane were nearly 60 kDa obtained using the DAB kit, whereas no specific bands were detected in the supernatant of normal cells (Figure 4B). The results revealed that rescued viruses could be identified by PCV3 Cap mAb.

### Immunofluorescence Detection

**FIGURE 5:**
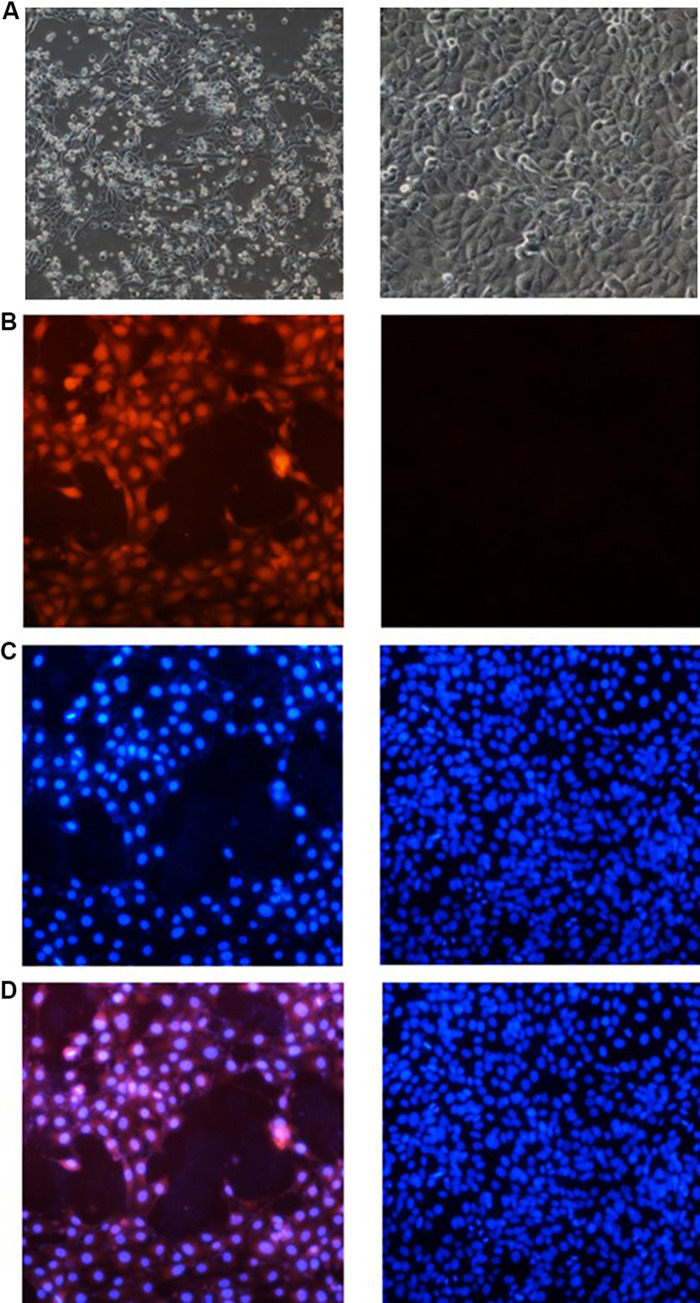
Experimental results of cellular immunofluorescence. **(A)** The morphology of cell. Morphology of normal 3D4/21 cells is shown on the right, and that of the cells inoculated with the rescued PCV3 after 72 h is on the left. **(B)** Cell immunofluorescence of D17 mAb to delve into the PCV3 infection with 3D4/21 cells. The data of D17 mAb is represented on the left side and the detection of normal cell is on the right. **(C)** Nuclear staining results of 3D4/21 cells. The left side represents the nuclear staining pictures in the left of panel **(B)**, and the right side is the relevant one in the right. **(D)** Results of image superposition. The cell pictures in the corresponding position of panels **(B,C)** are superimposed. The cell pictures of panel **(D)** comply with the cell positions of panels **(B,C)**.

The 3D4/21 cells were transfected with the cyclized PCV3 DNA, and the morphology was observed within 72 h. It was reported that the built infectious cloning of PCV3 may lead to the pathological variations in 3D4/21 (Figure 5A). By the immunofluorescence assay, viral antigens were identified by the PCV3 specific antibodies. D17 mAb was observed to be able to recognize PCV3 in cells, represented as red under a fluorescence microscope (Figure 5B), while no fluorescence was exhibited by control cells transfected in parallel as the mock control. The results revealed the presence of rescued PCV3 as determined.

The nuclei of PCV3-infected cells and normal cells were stained with a DAPI kit (Beyotime, Shanghai, China). The results of nuclear staining are presented in Figure 5C. By overlapping the pictures of the same location (Figure 5D), the PCV3 Cap was reported to be primarily expressed in the nucleus of cells.

### Viral Titration

**FIGURE 6:**
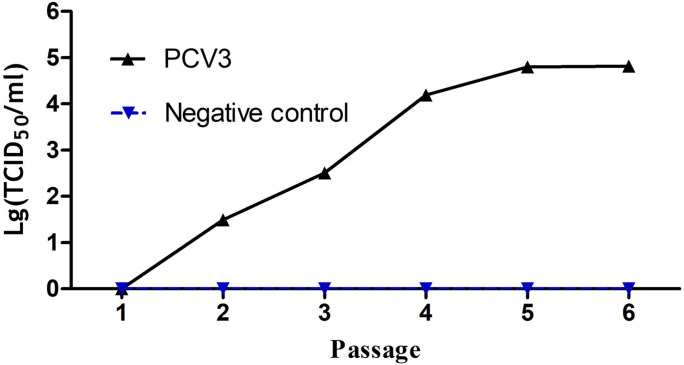
Growth curves of the cloned virus strains after a series of passages. The virus titers increased with the number of passages. The virus titers strikingly increased since the second passage and reached up to 10^4.8^ TCID_50_/ml at the sixth passage.

To assess the replication capabilities of the rescued PCV3 virus. 3D4/21 cells were inoculated the PCV3 that has been constructed from above for six passages. The virus titers increased during the second passage and reached up to 10^–4.8^ TCID_50_/ml at the sixth passage (Figure 6).

### Rescued PCV3 Virus Pathogenicity Analysis

**FIGURE 7:**
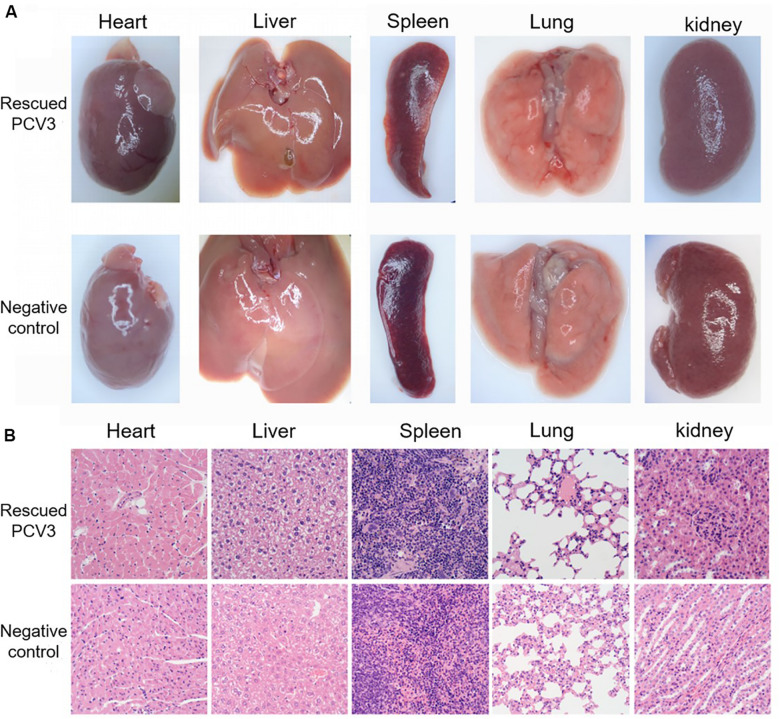
Clinical symptoms and histopathological analysis of organs in the PCV3-inoculated KM mice. **(A)** Mouse tissue anatomy. There were no noticeable differences between KM mice inoculated with PCV3 and normal KM mice in the morphology of tissue changes. **(B)** Tissue section results. The heart, liver, spleen, lung and kidney sections from no PCV3-inoculated KM mice exhibited normal morphology. Lung lesions from PCV3-inoculated KM mice showed local alveolar wall vascular dilatation and interstitial pneumonia, and no significant changes in other tissues.

During the experiment, no clinical variations were observed in the control group of mice. On the third day after infection, the infected group of mice was slightly depressed. Individual mice were reluctant to move, and no other symptoms occurred apparently. The tissues and organs of the control group and the experimental group were basically normal, and no significant variations were observed (Figure 7A).

Histopathological examination showed slight lesions in the tissues and organs of the control mice. In the mice in the infected group, significant changes in lung and heart tissues, the alveolar epithelial cells were proliferated in the local area of the lungs, and congestion took place at the edge of the local lobules, and other tissues varied slightly (Figure 7B).

### PCV3 Antigen Detection in Tissues

**FIGURE 8:**
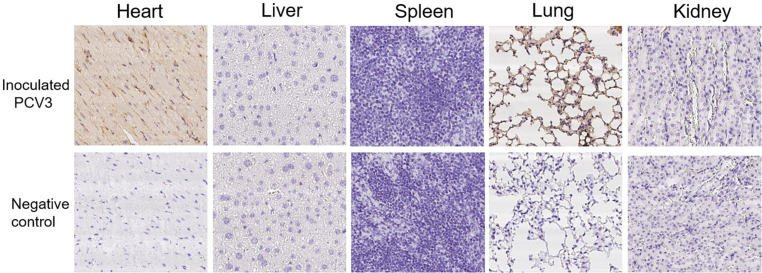
Immunohistochemical staining of organs and tissues of the PCV3-inoculated KM mice. No staining was observed in the heart, liver, spleen, lung, and kidney from no PCV3-inoculated KM mice, and no noticeable staining was observed in the liver, spleen and kidney from PCV3-inoculated KM mice. PCV3 antigen positive cells were brown. A considerable number of positive cells (arrow) for PCV3 antigen were observed in heart and lung tissues of the PCV3-inoculated KM mice.

For 6-week-old KM mice, lung, liver, kidney, spleen, and heart samples of all the mice subsequently underwent immunohistochemical staining of PCV3 antigen. Various tissues and organs developed similar distributions of PCV3 antigen. As shown in Figure 8, in the lung, the bronchial epithelial cell surface, interstitial vascular contents, alveolar exudate, dust cells, and septal cells all had robust positive reactions. Myocardial fibers showed varied positive reactions, and necrotic tissues and vascular contents had significantly positive reactions.

## Discussion

Previous studies have shown that PCV2 is the main pathogen causing PWMS and PDNS (Pogranichniy et al., 2002; Hamberg et al., 2007). However, since first reported in 2016 (Palinski et al., 2017), increasing epidemiological data (Ku et al., 2017; Stadejek et al., 2017; Zheng et al., 2017; Franzo et al., 2018; Fu et al., 2018; Kedkovid et al., 2018; Kim et al., 2018) suggested that PCV3 has been extensively distributed in pig farms worldwide. Researchers found that PCV3 can also cause PDNS (Yuzhakov et al., 2018), and is often infected in combination with PCV2 (Wang et al., 2019; Xia et al., 2019). As indicated, PCV3 was a potential pathogenic factor leading to an increase in the morbidity and mortality of swine; it has posed threats to the swine industry, causing significant economic losses. However, currently there is a lack of commercial vaccines for PCV3, the infection mechanism of PCV3 has not been clarified, and the gene structure and function of PCV3 have not been fully analyzed.

Separating and obtaining standard virus strains is the key to solving the above problems. Although researchers have constructed PCV3 infectious clones (Jiang H. et al., 2019), the methods for preparing PCV3 infectious clones mostly rely on the application of eukaryotic expression vectors, and use PK-15 cell line for virus packaging and rescue. It has been reported that there is a long-term presence in the PCV1 group (Denner and Mankertz, 2017), suggesting that there are certain risks in the current use of porcine cells and their use as animal models. Accordingly, PCV3 infectious clones prepared with PK-15 cells might be a mixed-gene PCV and an existed risk of contamination of foreign virus genomes. This study based on the clinical report that PCV3 can cause bronchial interstitial pneumonia in pigs (Palinski et al., 2017), 3D4/21 cell line was used for the first time as the packaging cell line of PCV3, confirming that PCV3 can be replicated and packaged in 3D4/21 cells. According to the genome structure of circovirus and its replication mechanism, it can be known that the circovirus itself has a replication initiation element, and the circovirus does not need to resort to an exogenous promoter when infecting the body. Therefore, according to the biological characteristics of circovirus, this research obtained PCV3 circular DNA *in vitro* to prepare PCV3 infectious clones. This will provide practical experience for the construction of other circular viruses and the construction of unknown circular virus infectious clones in the future.

The experimental results of cyclization demonstrated that large loops might be synthesized from the linearized PCV3 DNA by connecting several fragments, whereas there were also some end-to-end connections between the linearized PCV3 DNA, thereby leading to the formation of a closed loop. The PCV3 DNA containing several closed rings was transfected into cells, and the large rings in the PCV3 DNA might not be transcribed and expressed in cells or digested by intracellular digestive enzymes. In the present study, specific primers were designed, covering the PCV3 M1 and PCV3 L2 primers, the PCV3 complete genome amplification primers; PCV3 L3 primer was the reverse complementary sequence of PCV3 L2, and PCV3 M2 primer referred to the nucleotide sequence next to the primer binding site of PCV3 L2. The PCV3 Cap *Bam*HI and PCV3 Cap *Hin*dIII primers were designed as the reverse complement of the negative chain of PCV3 Cap gene. Subsequently, specific primers were conceived following the reverse complementary DNA sequence. The experimental results proved that cyclized PCV3 DNA was transfected into cells and then transcribed and duplicated after several blind passages.

In order to reduce the infection of PCV3 by the PCV1 virus carried by the pigs themselves, this study looked for other animal models to evaluate the pathogenicity of PCV3 infectious clones. Studies have shown that PCV3 has the ability of cross-host transmission of infected mice (Jiang S. et al., 2019), so this study uses SPF Kunming mice as a model of PCV3 infection. The results show that PCV3 can infect myocardium and lung tissue of Kunming mice, which is consistent with the current report that PCV3 can cause myocarditis in pigs (Phan et al., 2016). The molecule that PCV3 causes myocarditis in piglets still needs further exploration. Studies have shown that PCV3 can cause the expression level of IFN-γ to be up-regulated in piglet, and IFN-γ has been found to be related with viral myocarditis in the study of Coxsackie B3 virus (CVB3) causing myocarditis (Zhou et al., 2018). This provides a reference for further study on the mechanism of PCV3 induced myocarditis in piglets.

## Conclusion

In this study, our results demonstrated that infectious cloning of PCV3 were successfully obtained, rescued PCV3 virus could infect the heart muscle and lung of KM mice, and mAbs against PCV3 Cap were produced. The mentioned results can lay a theoretical and practical foundation for the accurate diagnosis of PCV3, the development of antibody drugs and subunit vaccines, as well as the analysis of relevant pathogenic sites. However, subsequent studies should still assess the significance of the findings to the biology and immunopathology of PCV3 infection.

## Data Availability Statement

The read sequences for this study were deposited to the NCBI Sequence Read Archive (SRA), accession number: MH107162.1.

## Ethics Statement

The animal study was reviewed and approved by the Ethics Committee of South China Agricultural University, Guangzhou, China.

## Author Contributions

ZJ, GZ, and ML designed the study. JW, MJ, and WB performed the experiments. YX and CL analyzed the data. ZJ and JW wrote the manuscript. ML and YX revised the manuscript. All authors reviewed the manuscript.

## Conflict of Interest

The authors declare that the research was conducted in the absence of any commercial or financial relationships that could be construed as a potential conflict of interest.
